# Supplementary material for: A midbrain GABAergic circuit constrains wakefulness in a mouse model of stress
Source: Nat Commun. 2024 Mar 28;15:2722. doi: 10.1038/s41467-024-46707-9 (PMC10978901; doi:10.1038/s41467-024-46707-9)
Supplement: Supplementary file 1 — Supplementary Information [file 41467_2024_46707_MOESM1_ESM.pdf]

A midbrain GABAergic circuit constrains wakefulness in a mouse model of stress

Supplementary Information

Supplementary information contains 16 supplementary figures.

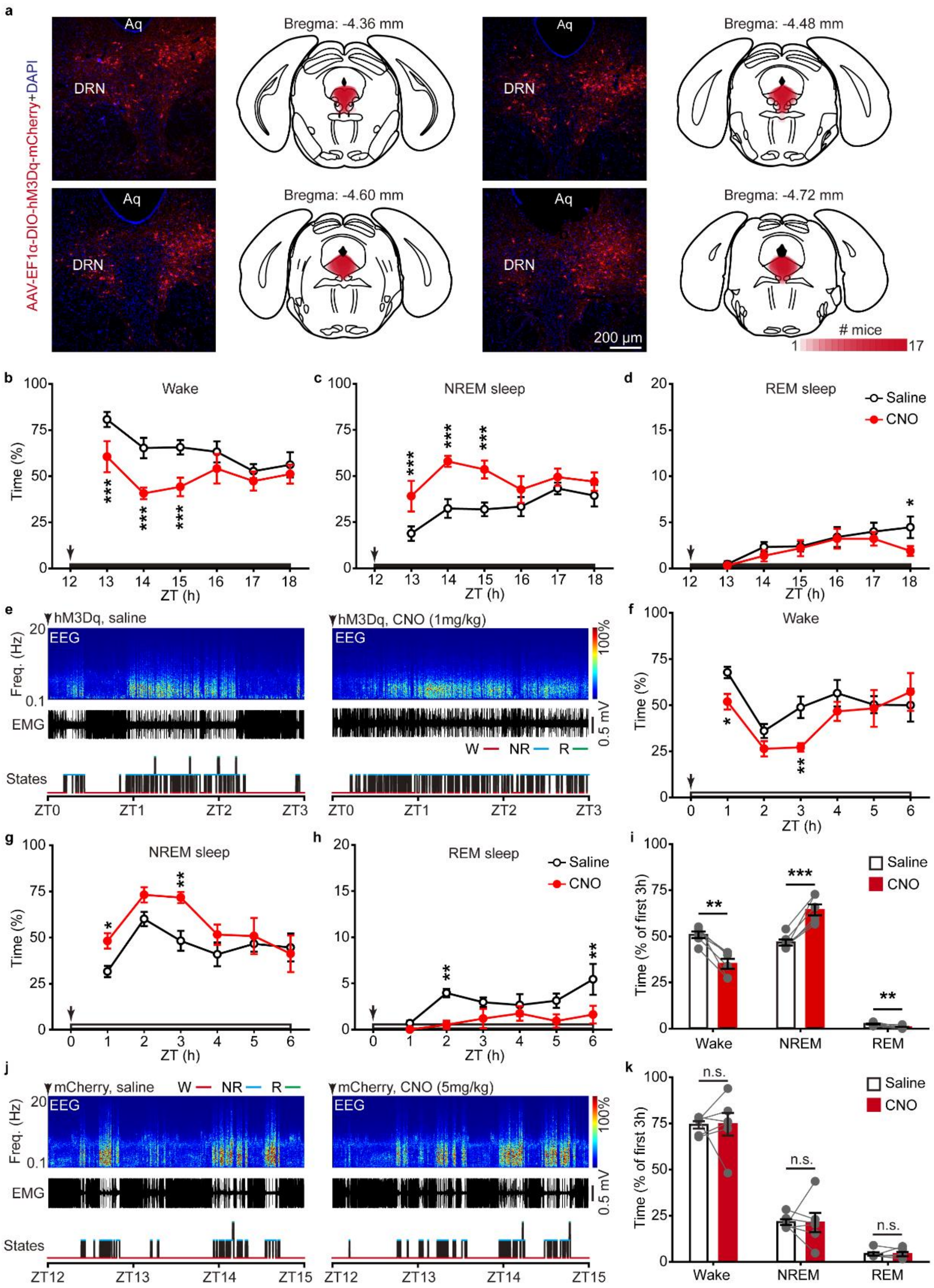

Supplementary Figure 1. Effects of chemogenetic activation of DRN^{GAD2} neurons on wakefulness/sleep. a, The infected area of hM3Dq-mCherry in the DRN and adjacent brain regions. Left, images showing coronal sections of DRN from GAD2-Cre mice injected with AAV-EF1α-DIO-hM3Dq-mCherry. Right, drawings of superimposed hM3Dq-mCherry expression in the DRN. Aq, aqueduct. **b** to **d**, Time course curves showing the time spent in wakefulness (**b**), NREM sleep (**c**), and REM sleep (**d**) of DRN^{GAD2}-hM3Dq mice following saline or CNO injection at the beginning of dark phase. Arrowheads indicate the time point of saline or CNO injection. n=11 mice, wake: $F_{1,50}$=32.719, P<0.001; NREM: $F_{1,50}$=44.737, P<0.001; REM: $F_{1,50}$=1.763, P=0.213. **e**, EEG power spectrogram, EMG traces, and hypnograms from a DRN^{GAD2}-hM3Dq mouse during 3h post saline (left) or CNO (1mg/kg, right) injection at the beginning of light phase. Freq., frequency; W, wake; NR: NREM; R: REM. **f** to **h**, similar to **b** to **d**, for saline or CNO was injected at the beginning of light phase. n=6 mice, wake: $F_{1,25}$=4.29, P=0.093; NREM: $F_{1,25}$=5.877, P=0.06; REM: $F_{1,25}$=17.681, P=0.008. **i**, Time spent in each state during the first 3h after saline of CNO injection. n=6 mice, wake: t_5=6.074, P=0.00175; NREM: t_5=6.924, P=0.000964; REM: t_5=4.997, P=0.00412. **j**, similar to **e**, for saline or CNO (5mg/kg) was injected into a DRN^{GAD2}-mCherry mouse. **k**, Time spent in each state of DRN^{GAD2}-mCherry mice during the first 3h after saline of CNO injection. *P<0.05, **P<0.01, ***P<0.001, n.s., not significant. Data were analyzed by two way repeated measure ANOVA following Bonferroni's multiple comparisons test (**b** to **d**, **f** to **h**) or two tailed paired t test (**i**, **k**) and presented as mean ± SEM.

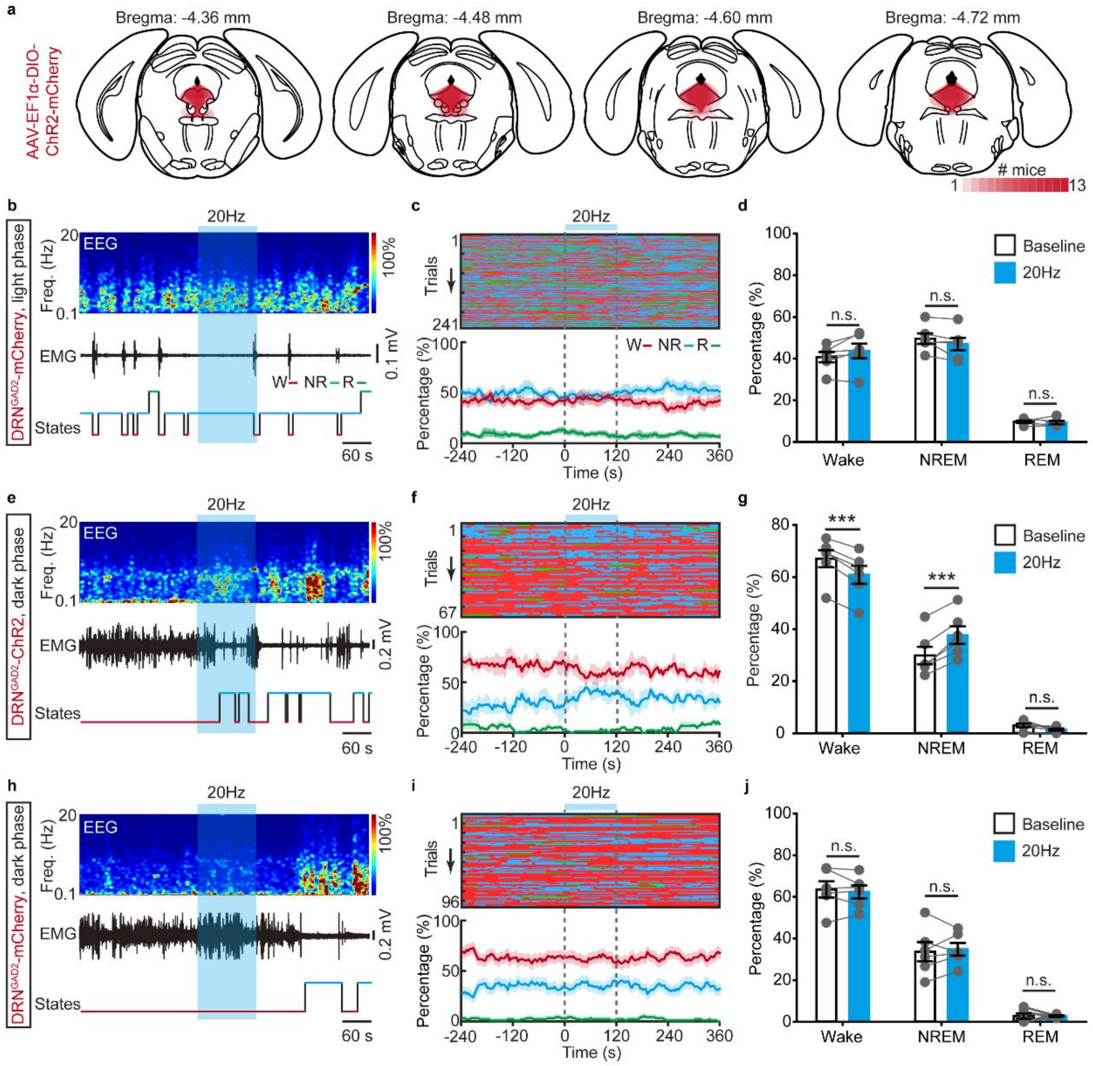

Supplementary Figure 2. Effects of optogenetic activation of DRN^{GAD2} neurons on wakefulness/sleep. **a**, Superimposed drawings showing the expression of ChR2-mCherry in the DRN of GAD2-Cre mice. **b**, **e**, **h**, EEG power spectrogram, EMG traces, and hypnograms showing optogenetic simulation in DRN^{GAD2}-mCherry mice during light phase (**b**) or dark phase (**h**) and in DRN^{GAD2}-ChR2 mice during dark phase (**e**). **c**, **f**, **i**, Top, brain states of recorded trials from DRN^{GAD2}-mCherry mice (**c**, **i**) and DRN^{GAD2}-ChR2 mice (**f**). Bottom, percentage of wake, NREM, or REM sleep around 20Hz stimulation of DRN^{GAD2}-mCherry mice (**c**, **i**) and DRN^{GAD2}-ChR2 mice (**f**). Shadings represent ± SEM. **d**, **g**, **j**, Quantification of time spent in each state 120s before and during optogenetic simulation of DRN^{GAD2}-mCherry mice (**d**, **j**) and DRN^{GAD2}-ChR2 mice (**g**). n=6 for DRN^{GAD2}-mCherry mice and n=7 for DRN^{GAD2}-ChR2 mice. For **g**, wake: t_6=10.7, P=3.93×10^{-5}; NREM: t_6=9.24, P=9.07×10^{-5}. ***P<0.001, n.s., not significant. Data were analyzed by two tailed paired t test (**d**, **g**, **j**) and presented as mean ± SEM.

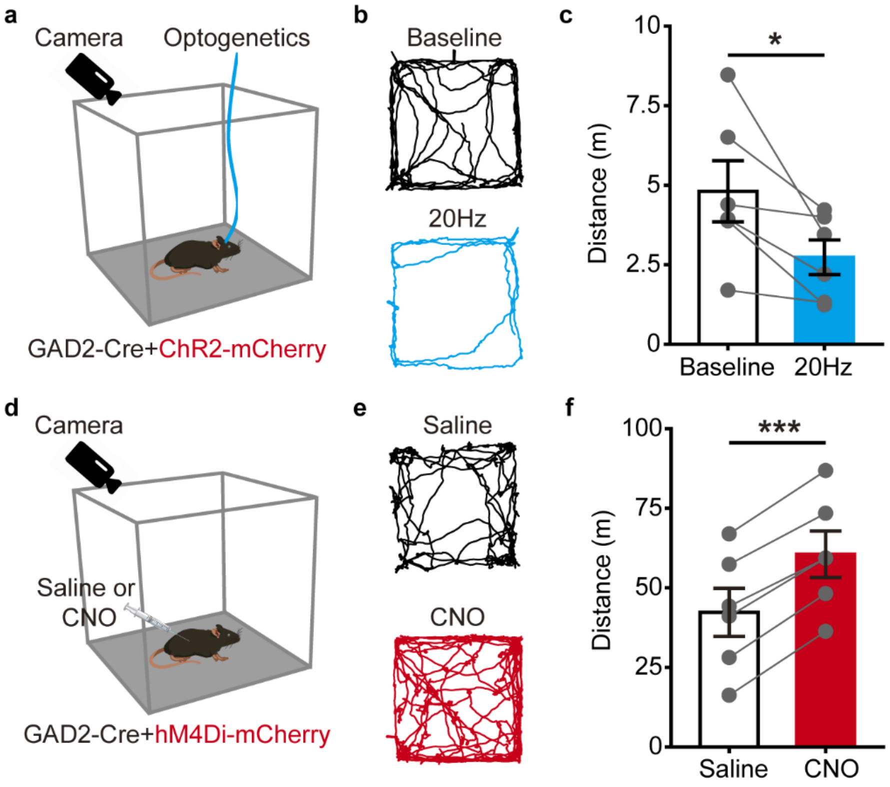

Supplementary Figure 3. Optogenetic activation or chemogenetic inhibition of DRN^{GAD2} neurons decreases or increases locomotor activity, respectively. a, **d**, Schematic experimental setup for optogenetic activation (**a**) or chemogenetic inhibition (**d**) of DRN^{GAD2} neurons in an open field. **b**, **e**, Representative video-tracked paths from a DRN^{GAD2}-ChR2 mouse in baseline and 20Hz optogenetic stimulation conditions (**b**) or a DRN^{GAD2}-hM4Di mouse following saline or CNO (2mg/kg) injection (**e**). **c**, **f**, Distance traveled of DRN^{GAD2}-ChR2 mice in baseline and 20Hz optogenetic stimulation conditions (**c**, n=6 mice, t_5=2.951, P=0.0319) or DRN^{GAD2}-hM4Di mice following saline or CNO injection (**f**, n=6 mice, t_5=20.364, P=5.28×10^{-6}). *P<0.05, ***P<0.001. Data were analyzed by two tailed paired t test (**c**, **f**) and presented as mean ± SEM.

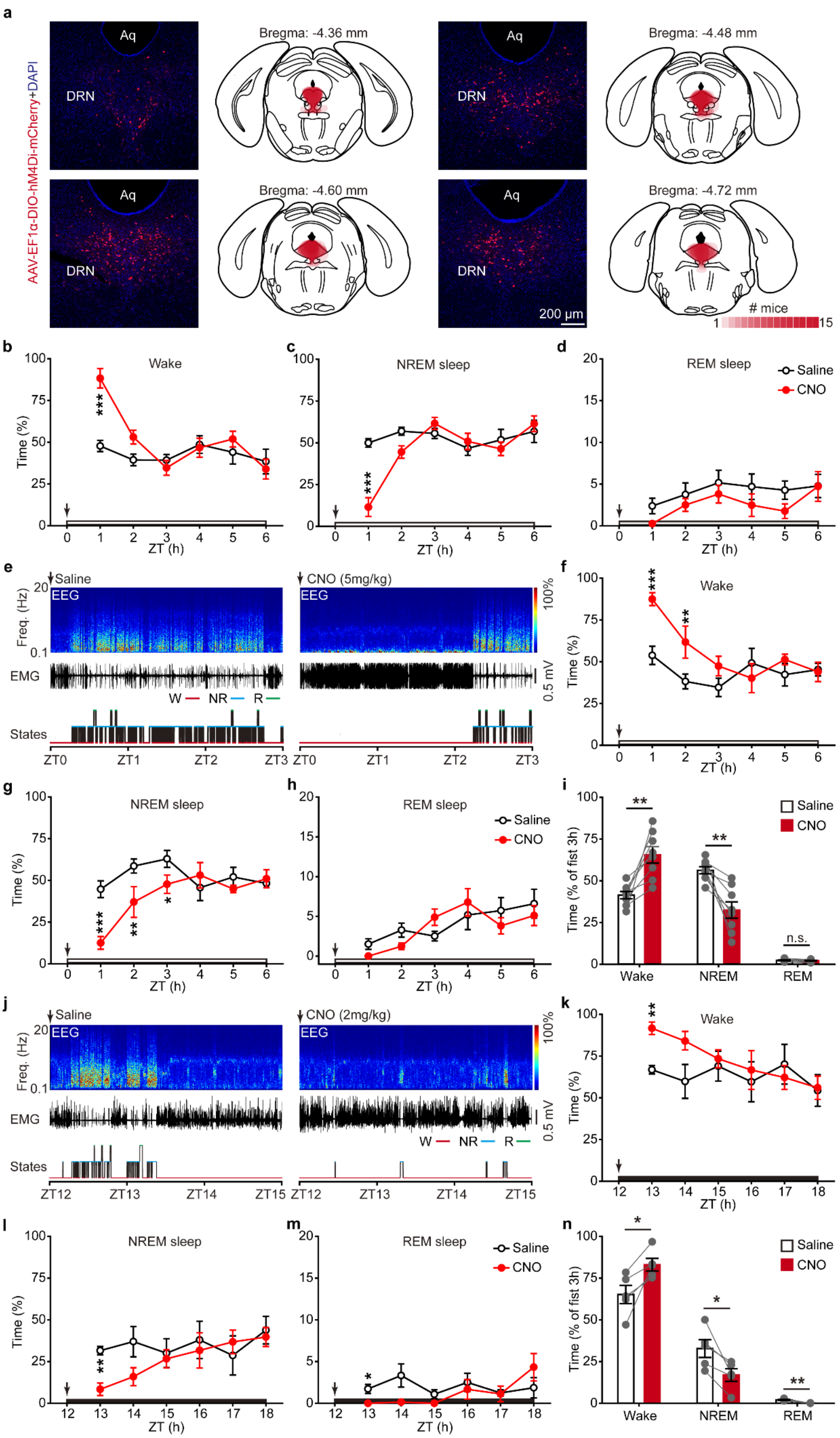
a
AAV-EF1α-DIO-hM4Di-mCherry+DAPI
Aq
DRN
Bregma: -4.36 mm
Bregma: -4.48 mm
Bregma: -4.60 mm
Bregma: -4.72 mm
200 μm
mice
1
15
b
Wake
Time (%)
ZT (h)
c
NREM sleep
d
REM sleep
Saline
CNO
e
Saline
CNO (5mg/kg)
EEG
Freq. (Hz)
EMG
0.5 mV
100%
W
NR
R
States
ZT0
ZT1
ZT2
ZT3
f
Wake
g
NREM sleep
h
REM sleep
i
Time (% of fist 3h)
Wake
NREM
REM
n.s.
j
CNO (2mg/kg)
ZT12
ZT13
ZT14
ZT15
k
Wake
l
NREM sleep
m
REM sleep
n

Supplementary Figure 4. Effects of chemogentic inhibition of DRN^{GAD2} neurons on wakefulness/sleep. a, The infected area of hM4Di-mCherry in the DRN and adjacent brain regions. Left, images showing coronal sections of DRN from GAD2-Cre mice injected with AAV-EF1α-DIO-hM4Di-mCherry. Right, drawings of superimposed hM4Di-mCherry expression in the DRN. Aq, aqueduct. **b** to **d**, Time course curves showing the time spent in wakefulness (**b**), NREM sleep (**c**), and REM sleep (**d**) of DRN^{GAD2}-hM4Di mice following saline or CNO (2mg/kg) injection at the beginning of light phase. Arrowheads indicate the time point of saline or CNO injection. n=7 mice, wake: $F_{1, 30}$=6.137, P=0.048; NREM: $F_{1, 30}$=6.214, P=0.047; REM: $F_{1, 30}$=4.321, P=0.083. **e**, EEG power spectrogram, EMG traces, and hypnograms from a DRN^{GAD2}-hM4Di mouse during 3h post saline or CNO (5mg/kg) injection at the beginning of light phase. Freq., frequency; W, wake; NR: NREM; R: REM. **f** to **h**, Similar to **b** to **d**, for CNO was used at 5mg/kg. n=8 mice, wake: $F_{1, 35}$=8.853, P=0.021; NREM: $F_{1, 35}$=9.751, P=0.017; REM: $F_{1, 35}$=0.605, P=0.462. **i**, Time spent in each state during the first 3h after saline of CNO injection. n=8 mice, wake: t_7=4.277, P=0.00367; NREM: t_7=4.348, P=0.00336; REM: t_7=0.96, P=0.369. **j**, Similar to **e**, for saline or CNO (2mg/kg) was injected at the beginning of dark phase. **k** to **m**, similar to **b** to **d**, for saline or CNO was injected at ZT12. n=5 mice, wake: $F_{1, 20}$=3.088, P=0.157; NREM: $F_{1, 20}$=2.874, P=0.167; REM: $F_{1, 20}$=4.973, P=0.09. **n**, Time spent in each state during the first 3h after saline of CNO injection at ZT12. n=5 mice, wake: t_4=3.763, P=0.0197; NREM: t_4=3.521, P=0.0244; REM: t_4=7.352, P=0.00182. *P<0.05, **P<0.01, ***P<0.001, n.s., not significant. Data were analyzed by two way repeated measure ANOVA following Bonferroni's multiple comparisons test (**b** to **d**, **f** to **h**, **k** to **m**) or two tailed paired t test (**i**, **n**) and presented as mean ± SEM.

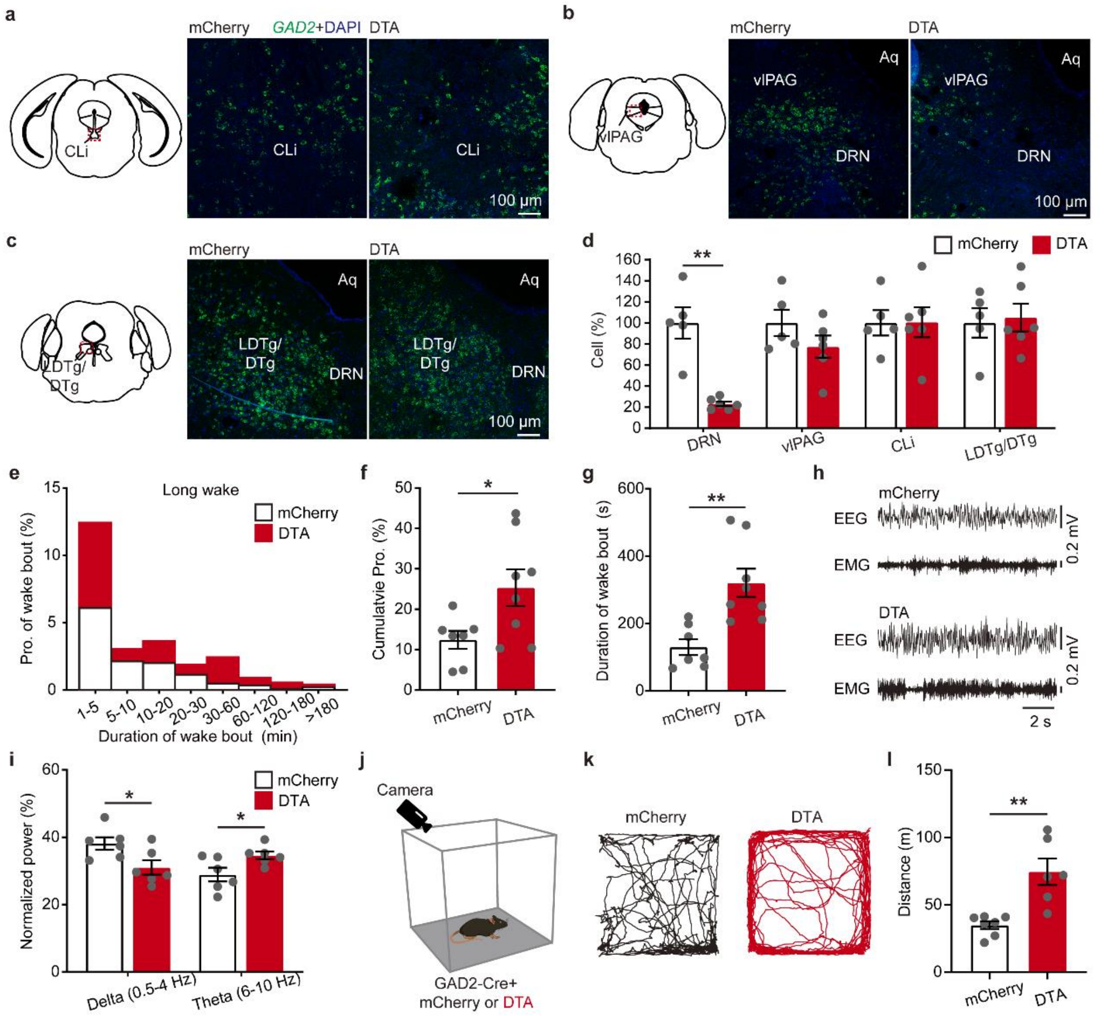

Supplementary Figure 5. Ablation of DRN^{GAD2} neurons prolongs wake bout, alters EEG activity, and increases locomotor activity. a to **c**, Representative images showing *GAD2*-positive neurons in the DRN and adjacent brain regions in mCherry control or DTA mice. Cli, caudal linear nucleus of the raphe; vlPAG, ventrolateral periaqueductal gray; LDTg/DTg, laterodorsal tegmental nucleus/dorsal tegmental nucleus; Aq, aqueduct. **d**, Quantifications of *GAD2*-positive neurons in mCherry and DTA group. n=5 for mCherry mice and n=6 for DTA mice, DRN: P=0.004. **e**, Probability of long-bout (>1min) wake of DTA mice or mCherry mice. Pro., probability. **f**, Analysis of cumulative probability of long-bout wake. n=7 for mCherry mice and n=8 for DTA mice, t_{13}=2.43, P=0.0303. **g**, The duration of wake bout of DTA mice or mCherry mice. n=7 for mCherry mice and n=8 for DTA mice, t_{13}=3.809, P=0.00217. **h**, Raw EEG and EMG traces of wakefulness in DTA and mCherry mice. **i**, Normalized EEG delta and theta power density of wakefulness during the dark phase. n=6 mice for each group, Delta: t_{10}=2.502, P=0.0313; Theta: t_{10}=2.464, P=0.0334. **j**, Schematic experimental setup for testing the effects of ablation of DRN^{GAD2} neurons on locomotor activity. **k**, Representative video-tracked paths from a mCherry mouse and a DTA mouse. **l**, Distance traveled in the open field over a 10min period. n=7 for mCherry mice and n=6 for DTA mice, t_{11}=4.138, P=0.00165. *P<0.05, **P<0.01. Data were analyzed by Mann-Whitney rank sum test (**d**), two tailed unpaired t test (**f**, **g**, **i**, **l**) and presented as mean ± SEM.

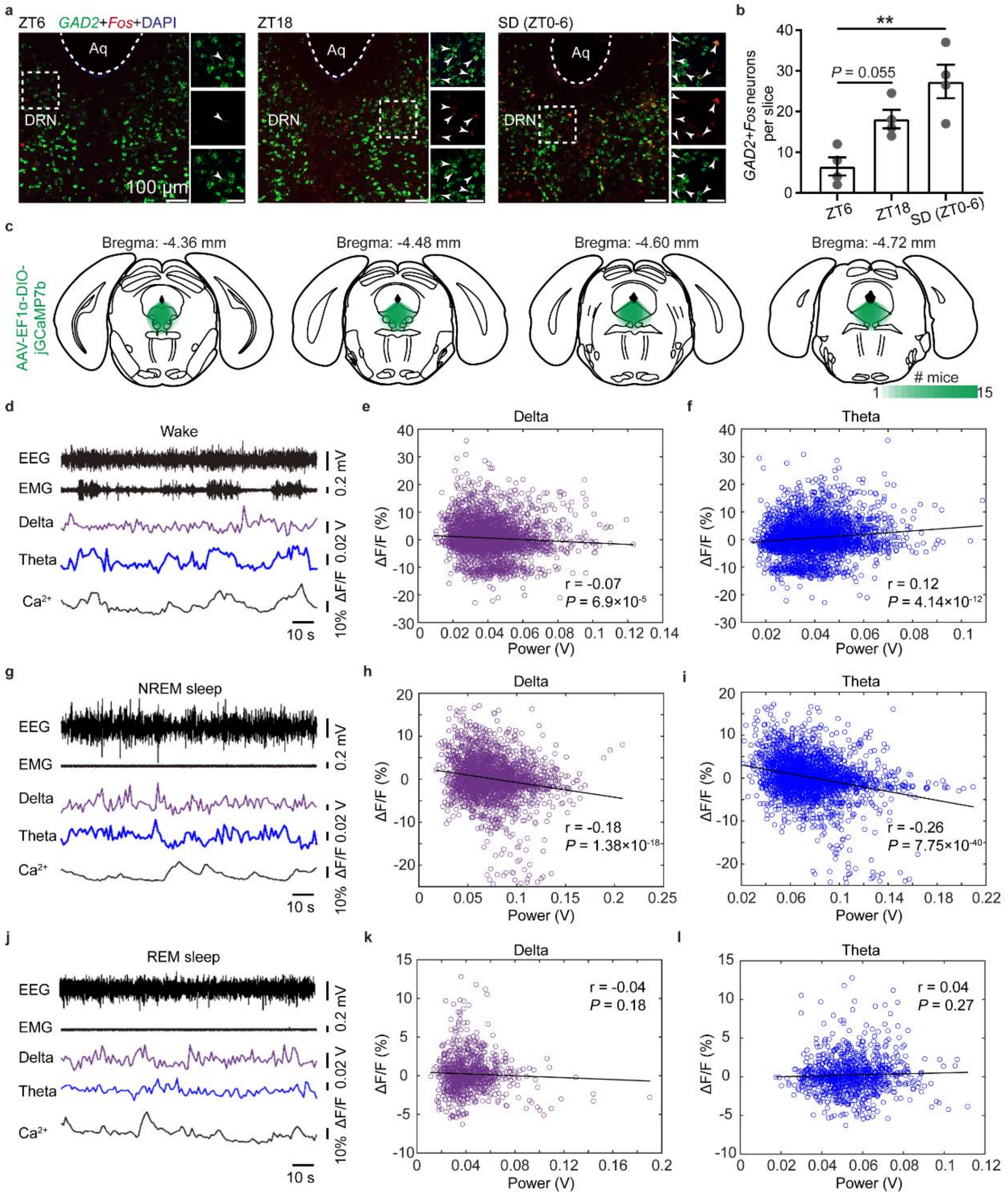

Supplementary Figure 6. *Fos* expression pattern in DRN^{GAD2} neurons at distinct time points of the light/dark cycle, correlation between Ca^{2+} activities of DRN^{GAD2} neurons and specific components of EEG activity. a, Representative images showing *Fos* expression in DRN^{GAD2} neurons at ZT6, ZT18, and after 6h sleep deprivation (SD, ZT0-6). Boxed areas in the left panels are enlarged in the right panels. Arrowheads indicate *Fos*- and *GAD2*-double-positive neurons. Scale bars in the right panels are 50µm. Aq, aqueduct. **b**, Quantitative analysis *Fos* expression in DRN^{GAD2} neurons at ZT6, ZT18 and SD. n=4 mice per group, $F_{2,9}$=12.124, ZT6 vs ZT18: *P*=0.055, ZT6 vs SD: *P*=0.002. **c**, Superimposed drawings showing the expression of jGCaMP7b in the DRN. **d**, **g**, **j**, Representative raw EEG traces, EEG delta power, EEG theta power and Ca^{2+} fluorescence traces during wakefulness (**d**), NREM sleep (**g**), and REM sleep (**j**). **e**, **h**, **k**, Correlations between EEG delta power and Ca^{2+} activities of DRN^{GAD2} neurons during spontaneous wakefulness (**e**), NREM sleep (**h**), and REM sleep (**k**). **f**, **i**, **l**, Correlations between EEG theta power and Ca^{2+}

activities of DRN^{GAD2} neurons during spontaneous wakefulness (**f**), NREM sleep (**i**), and REM sleep (**l**). Each dots represent a 1s epoch. Black lines are the best linear fit, r is the correlation coefficient. $^{**}P<0.01$. Data were analyzed by one way ANOVA followed by Tukey's post hoc test (**b**) and presented as mean ± SEM.

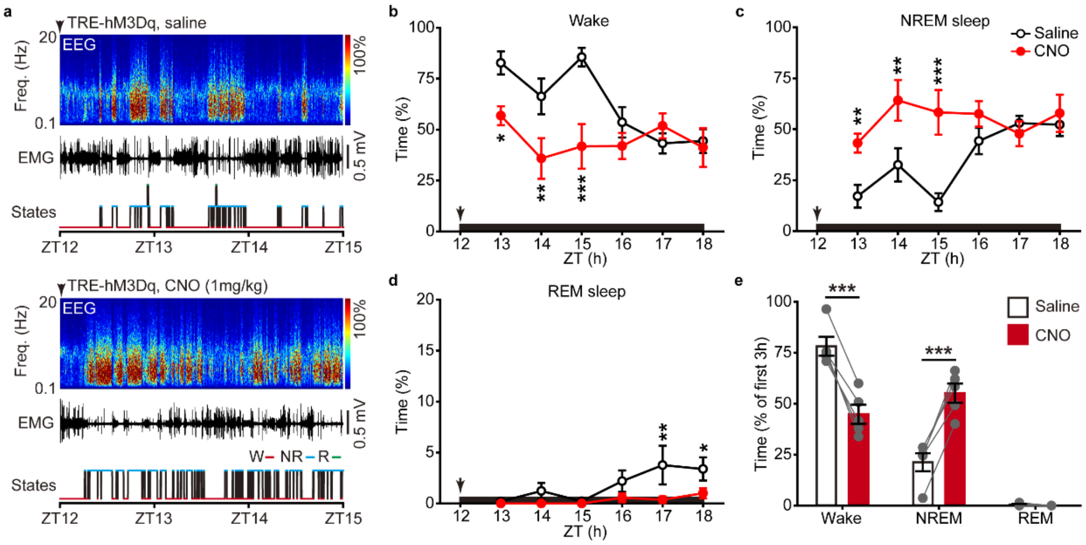

Supplementary Figure 7. Chemogenetic activation of acute restraint stress activated-DRN^{GAD2} neurons decreases wakefulness. a, EEG power spectrogram, EMG trances, and hypnograms from a DRN^{GAD2}-TRE-hM3Dq mouse during 3h post saline or CNO (1mg/kg) injection at the beginning of dark phase. Freq., frequency; W, wake; NR: NREM; R: REM. **b** to **d**, Time course curves showing the time spent in wakefulness (**b**), NREM sleep (**c**), and REM sleep (**d**) following saline or CNO injection at the beginning of dark phase. Arrowheads indicate the time point of saline or CNO injection. n=5 mice, wake: $F_{1,\ 20}$=24.944, P=0.008; NREM: $F_{1,\ 20}$=28.443, P=0.006; REM: $F_{1,\ 20}$=6.281, P=0.066. **e**, Time spent in each state during the first 3h after saline of CNO injection. n=5 mice, wake: t_4=10.976, P=3.91×10^{-4}; NREM: t_4=11.467, P=3.3×10^{-4}. *P<0.05, **P<0.01, ***P<0.001. Data were analyzed by two way repeated measure ANOVA following Bonferroni's multiple comparisons test (**b** to **d**) or two tailed paired t test (**e**) and presented as mean ± SEM.

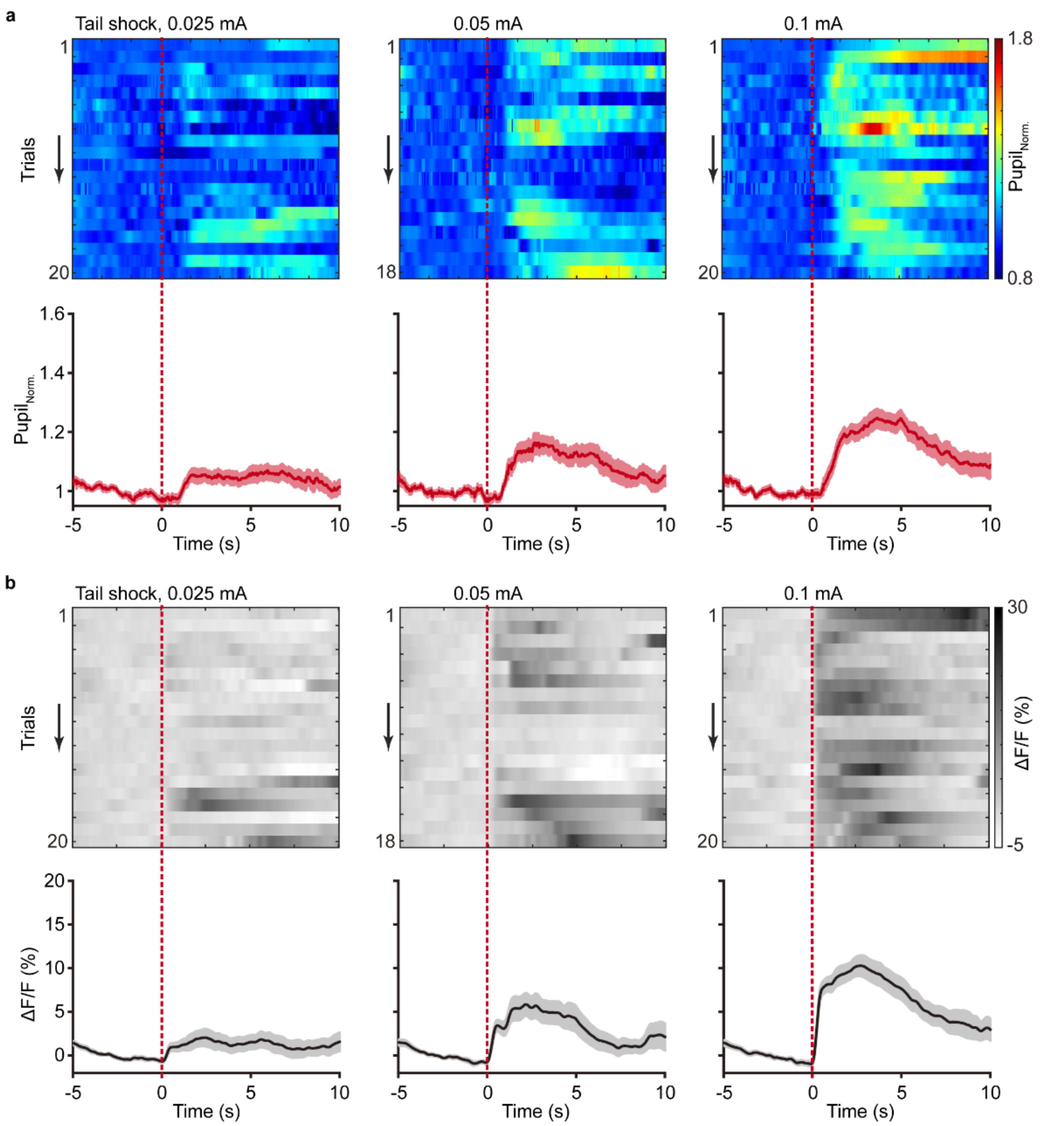

Supplementary Figure 8. Tail shock increases pupil size and Ca^{2+} activities of DRN^{GAD2} neurons. **a**, **b**, Heatmaps (top) and average traces (bottom) of pupil size (**a**) or Ca^{2+} activities (**b**) of DRN^{GAD2} neurons aligned to different amplitude of tail shock onset (vertical red line). Pupil$_{Norm.}$, normalized pupil size. Shadings represent ± SEM.

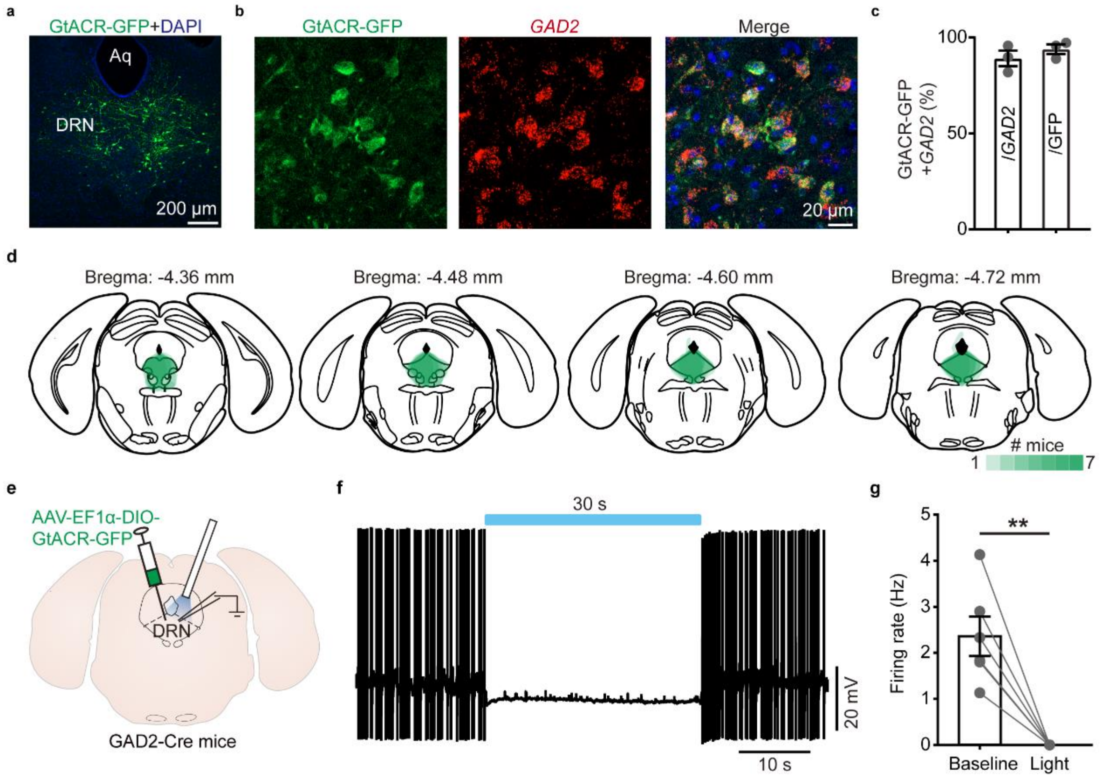

Supplementary Figure 9. Patch-clamp recording verifies the functional expression of GtACR-GFP in DRN^{GAD2} neurons. **a**, **b**, Representative images showing the expression of GtACR-GFP in the DRN (**a**) and the colocalization of GtACR-GFP with *GAD2* mRNA (**b**). Aq, aqueduct. **c**, Quantification of colocalization of between GtACR-GFP and *GAD2.* n=3 mice. **d**, Superimposed drawings showing the expression of GtACR-GFP in the DRN. **e**, Schematic diagram of experiment to examine the functional expression of GtACR-GFP using whole-cell patch-clamp recording. **f**, Example trace from a recorded GtACR-GFP-positive DRN^{GAD2} neuron. 30s constant blue laser stimulation abolished the spontaneous firing of action potentials of recorded neuron. **g**, Firing rate of action potentials in baseline conditions and during 30s blue laser stimulation. n=6 cells from 3 mice, t_5=5.48, P=0.00276. **P<0.01. Data were analyzed by two tailed paired t test (**g**). Data (**c**, **g**) are presented as mean ± SEM.

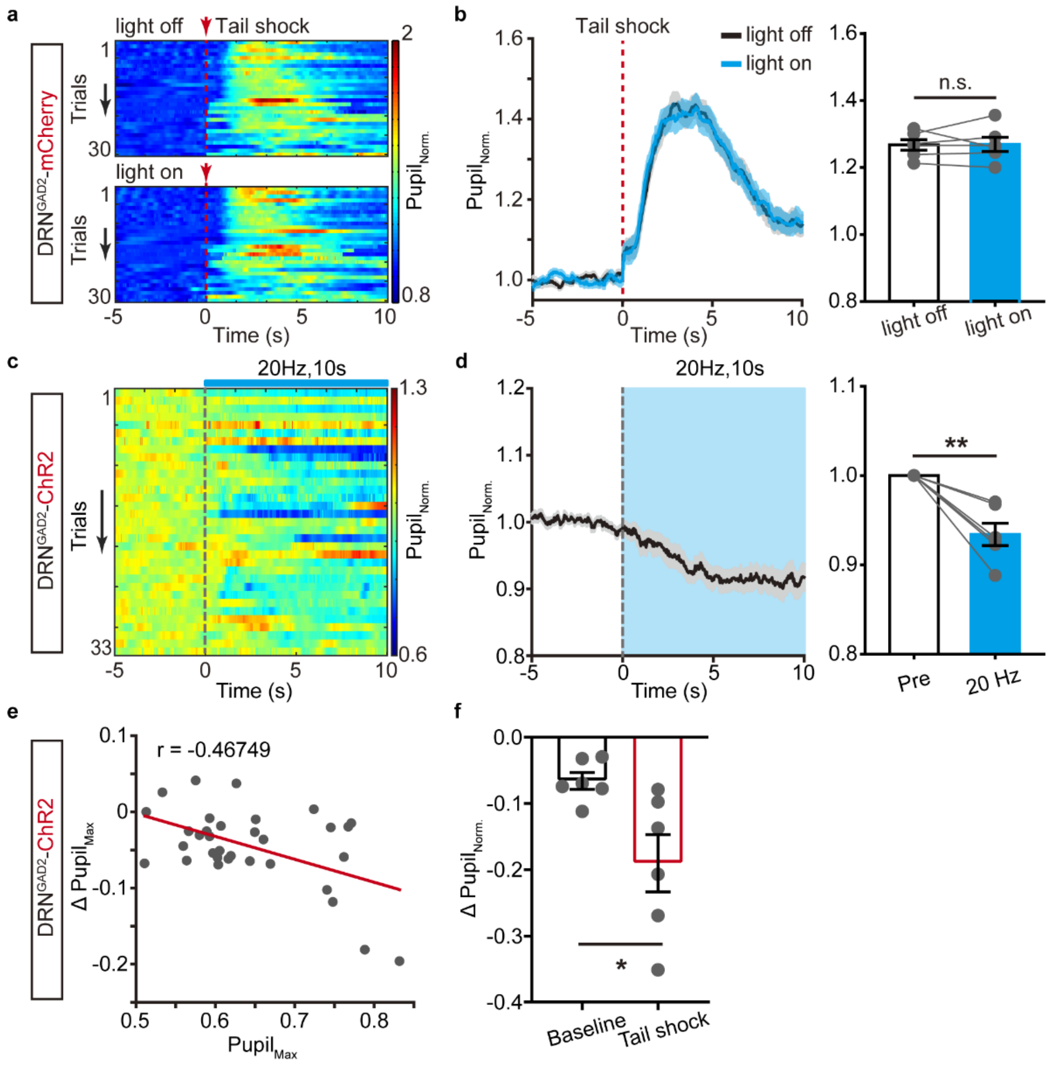

Supplementary Figure 10. Pupil size decrease following DRN^{GAD2} neurons activation depends on the baseline level of arousal. **a**, Heatmaps showing tail shock-induced changes of pupil size in DRN^{GAD2}-mCherry mice. Pupil$_{Norm.}$, normalized pupil size. **b**, Laser stimulation has no obvious effects on tail shock-induced changes of pupil size. n=6 mice, t_5=0.117, P=0.991. Shading represents ± SEM. **c**, Heatmap of all pupil trials from DRN^{GAD2}-ChR2 mice around 20Hz,10s optogenetic stimulation. **d**, Effects of optogenetic activation of DRN^{GAD2} neurons on pupil size. n=6 mice, t_5=5.209, P=0.00344. Shading represents ± SEM. **e**, Scatter plot showing the influence of baseline pupil size on activation of DRN^{GAD2} neurons-induced decrease of pupil size. Pupil$_{Max}$, max pupil size. **f**, The change of pupil size following optogenetic activation of DRN^{GAD2} neurons in baseline conditions and in tail shock conditions. n=6 mice for each group, t_{10}=2.756, P=0.0203. *P<0.05, **P<0.01, n.s., not significant. Data were analyzed by two tailed paired t test (**b**, **d**) or two tailed unpaired t test (**f**) and presented as mean ± SEM.

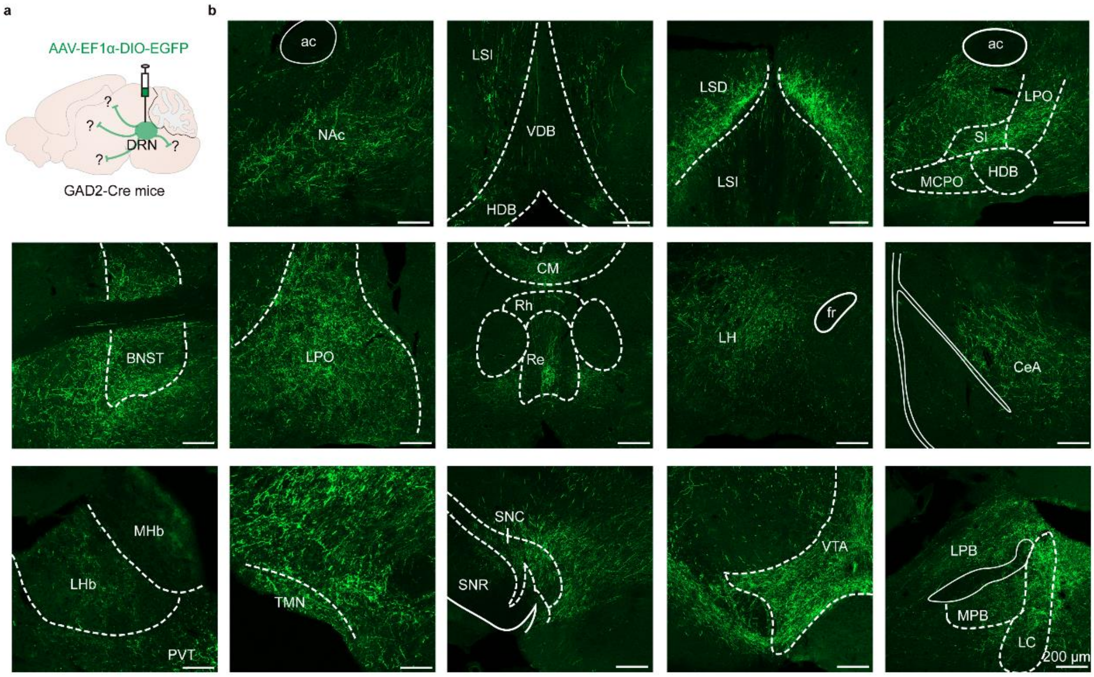

Supplementary Figure 11. Axonal projection pattern of DRN^{GAD2} neurons across the whole brain. a, Schematic of viral injection. AAV-EF1α-DIO-EGFP was injected into the DRN in GAD2-Cre mice. **b**, Axonal terminals distribution of DRN^{GAD2} neurons in different brain regions. Results were repeated in 4 mice.

Abbreviations: ac, anterior commissure; NAc, nucleus accumbens; LSI, lateral septal nucleus, intermediate part; VDB: nucleus of the vertical limb of the diagonal band; HDB, nucleus of the horizontal limb of the diagonal band; LSD: lateral septal nucleus, dorsal part; LPO: lateral preoptic area; SI: substantia innominate; MCPO: magnocellular preoptic nucleus; BNST, bed nucleus of the stria terminalis; CM, central medial thalamic nucleus; Rh, rhomboid thalamic nucleus; Re, reuniens thalamic nucleus; LH, lateral hypothalamus; fr, fasciculus retroflexus; CeA: central amygdaloid nucleus; MHb, medial habenular nucleus; LHb, lateral habenular nucleus; TMN, tuberomammillary nucleus; SNR, substantia nigra, reticular part; SNC, substantia nigra, compact part; VTA: ventral tegmental area; LPB: lateral parabrachial nucleus; MPB: medial parabrachial nucleus; LC: locus ceruleus.

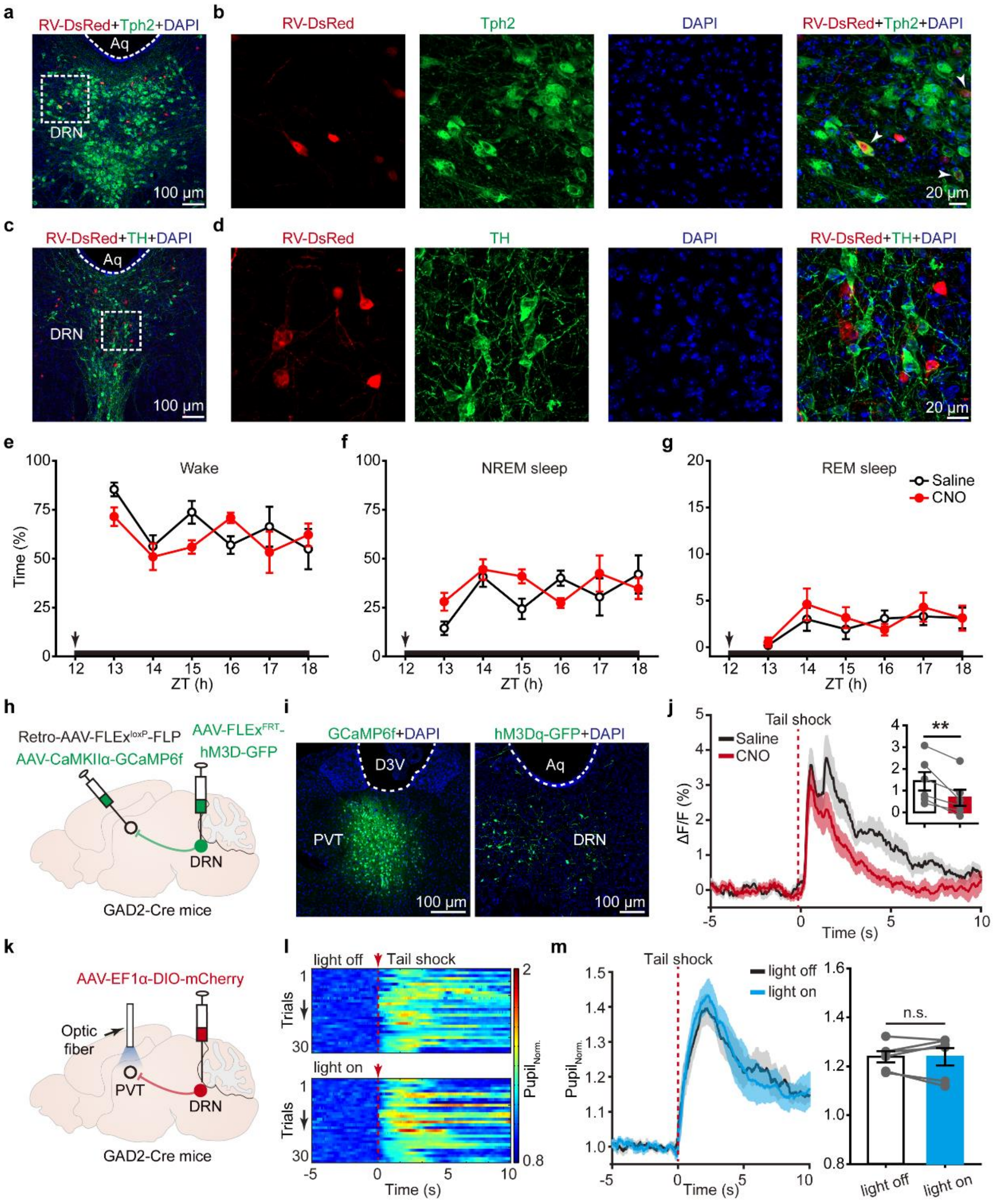

Supplementary Figure 12. Identification of monosynaptic inputs from the DRN to the PVT, time course cures of wakefulness/sleep following chemogenetic activation of the DRN^{GAD2}-PVT circuit, effects of chemogenetic activation of the DRN^{GAD2}-PVT circuit on tail shock-induced changes of PVT activity, and control experiments for optogenetic activation of the DRN^{GAD2}-PVT circuit. a to **d**, Representative images showing co-labeling of dsRed-positive neurons with Tph2-positive (**a**, **b**) and TH-positive (**c**, **d**) neurons in the DRN. **b**, **d**, Amplified view of boxed area in (**a**) or (**c**). Aq, aqueduct. **e** to **g**, Time course curves showing the time spent in wakefulness (**e**), NREM sleep (**f**), and REM sleep (**g**) of DRN^{GAD2}-PVT-hM3Dq mice following saline or CNO injection at the beginning of dark phase. Arrowheads indicate the time point of saline or CNO injection. n=6 mice. wake: $F_{1, 25}$=5.297, P=0.168; NREM: $F_{1, 25}$=2.26, P=0.193; REM: $F_{1, 25}$=3.681, P=0.113. **h**, Schematic of viral injection. A mixture of Retro-AAV-FLEx^{loxp}-FLP and

AAV-CaMKIIα-GCaMP6f was injected into the PVT and AAV-FLEx^{FRT}-hM3Dq-GFP was injected into the DRN. **i**, Representative images showing hM3Dq-GFP and GCaMP6f expression in the DRN (left) and PVT (right), respectively. D3V, dorsal third ventricle. **j**, Chemogenetic activation of DRN^{GAD2}-PVT circuit reduced the Ca^{2+} signals of PVT neurons evoked by tail shock. n=6 mice, t_5=4.079, P=0.00955. Shading represents ± SEM. **k**, Schematic diagram of optogenetic activation of DRN^{GAD2}-PVT pathway in DRN^{GAD2}-mCherry mice. **l**, Heatmaps showing tail shock-induced changes of pupil size. Pupil$_{Norm.}$, normalized pupil size. **m**, Laser stimulation of DRN^{GAD2}-PVT pathway has no obvious on tail shock-induced changes of pupil size in DRN^{GAD2}-mCherry mice. n=6 mice, t_5=0.00241, P=0.998. Shading represents ± SEM. **P<0.01, n.s., not significant. Data were analyzed by two way repeated measure ANOVA following Bonferroni's multiple comparisons test (**e** to **g**) or two tailed paired t test (**j**, **m**) and presented as mean ± SEM.

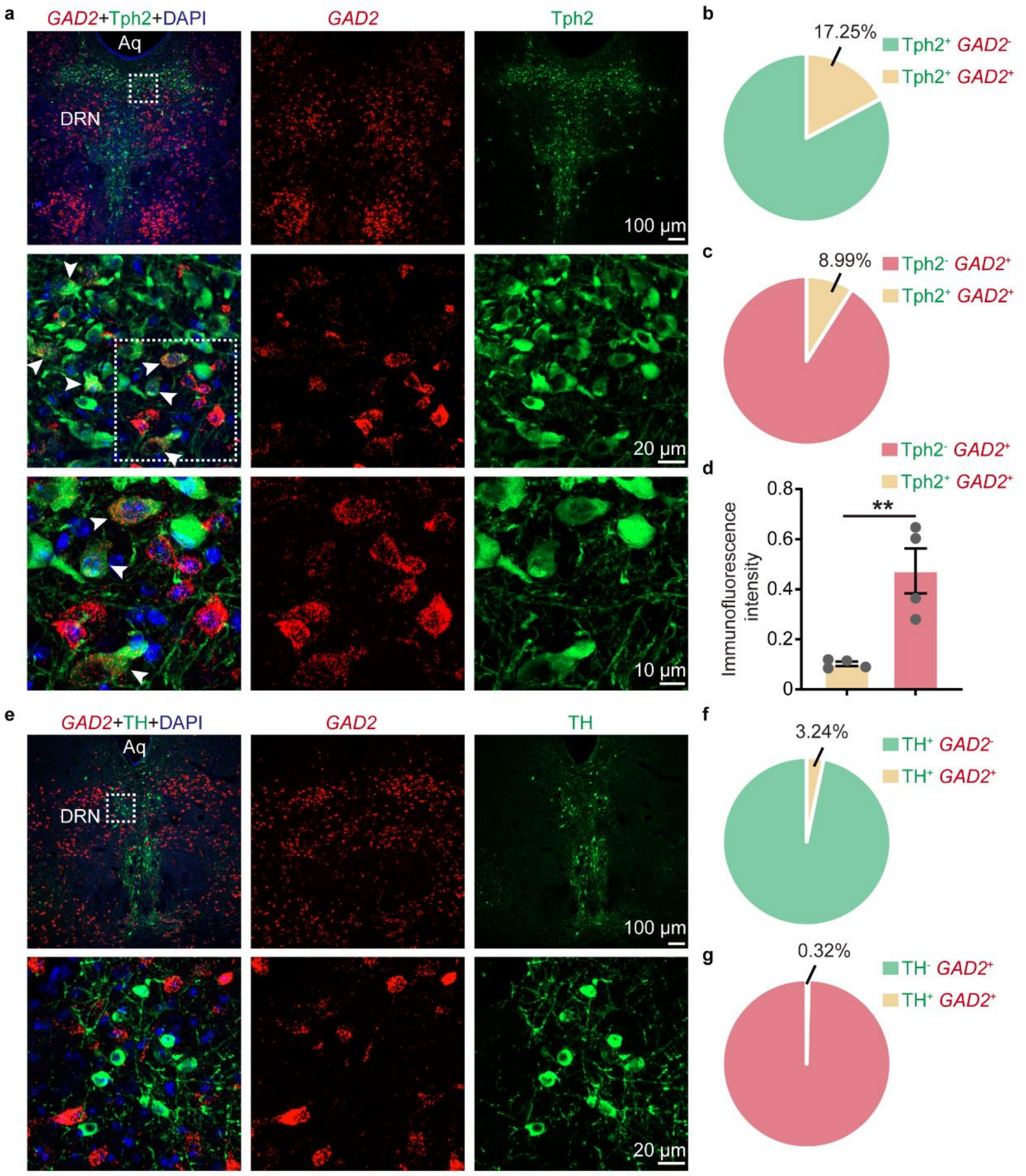

Supplementary Figure 13. Characterization of the expression pattern of *GAD2*, Tph2, and TH neurons in the DRN in wild type mice. a, Representative images showing the expression of *GAD2* and Tph2 neurons in DRN. Dashed boxes in the top panels are enlarged at the bottom panels. White arrowheads mark examples of *GAD2*- and Tph2-double-positive neurons. Aq, aqueduct. **b**, **c**, Pie charts showing the percentage of *GAD2*- and Tph2-double-positive neurons (yellow) in all Tph2 neurons (**b**, green) or *GAD2* neurons (**c**, red). n=4 mice. **d**, Quantification of immunofluorescence intensity of *GAD2* mRNA in Tph2 or non-Tph2 neurons. n=4 mice for each gropu, t_6=4.125, P=0.00618. **e**, Images showing the expression of *GAD2* and TH neurons in the DRN. **f**, **g**, Pie charts showing the percentage of *GAD2*- and TH-double-positive neurons (yellow) in all TH neurons (**f**, green) or *GAD2* neurons (**g**, red). n=4 mice. $^{**}P<0.01$. Data were analyzed by two tailed unpaired *t* test (**d**) and presented as mean ± SEM.

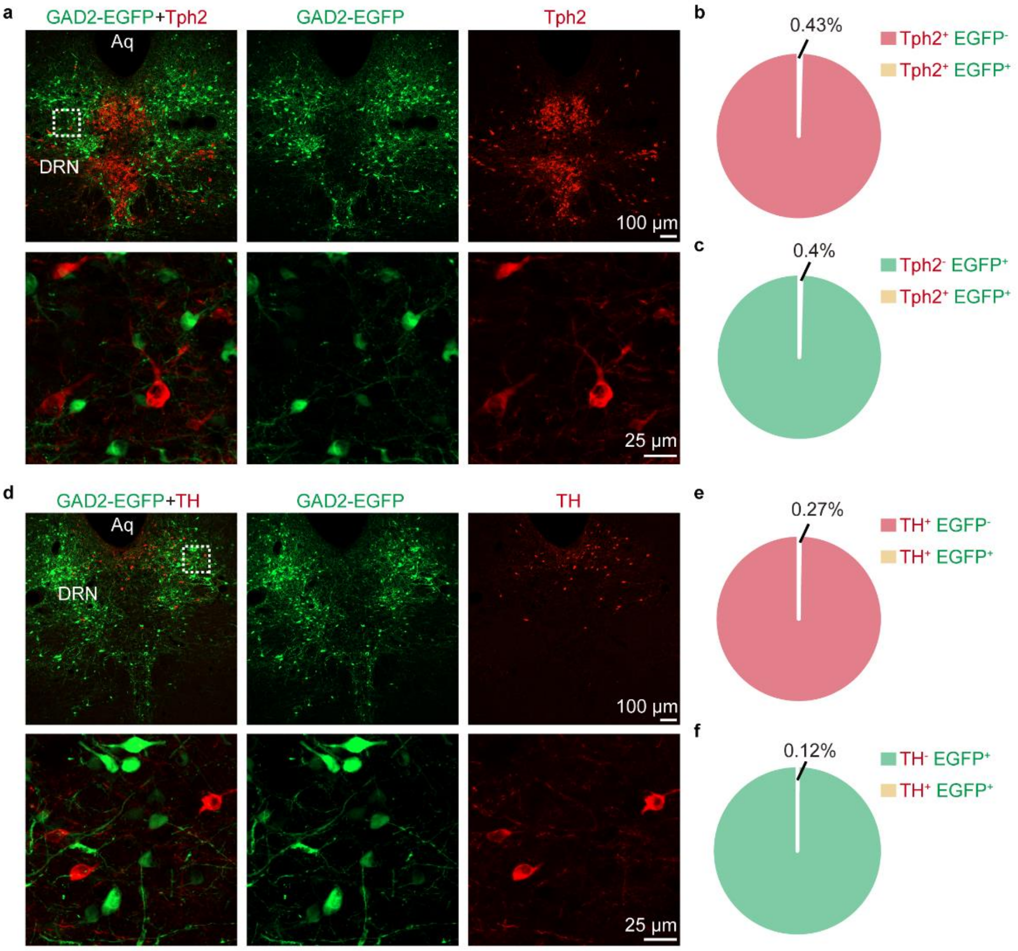

Supplementary Figure 14. Characterization of the expression pattern of EGFP-labeled GAD2 neurons, TpH2, and TH neurons in the DRN in GAD2-Cre mice. **a**, Representative images showing the expression of EGFP-labeled GAD2 neurons and Tph2 neurons in the DRN. GAD2 neurons was labeled with EGFP by injecting AAV-EF1α-DIO-EGFP into the DRN of GAD2-Cre mice. Bottom panels are enlarged view of the dashed box area in the top panels. Aq, aqueduct. **b**, **c**, Pie charts showing the percentage of EGFP- and Tph2-double positive neurons (yellow) in all Tph2 neurons (**b**, green) or EGFP neurons (**c**, red). n=4 mice. **d**, Images showing the expression of EGFP-labeled GAD2 neurons and TH neurons in the DRN. **e**, **f**, Pie charts showing the percentage of EGFP- and TH-double positive neurons (yellow) in all TH neurons (**e**, red) or EGFP-labeled neurons (**f**, green). n=4 mice.

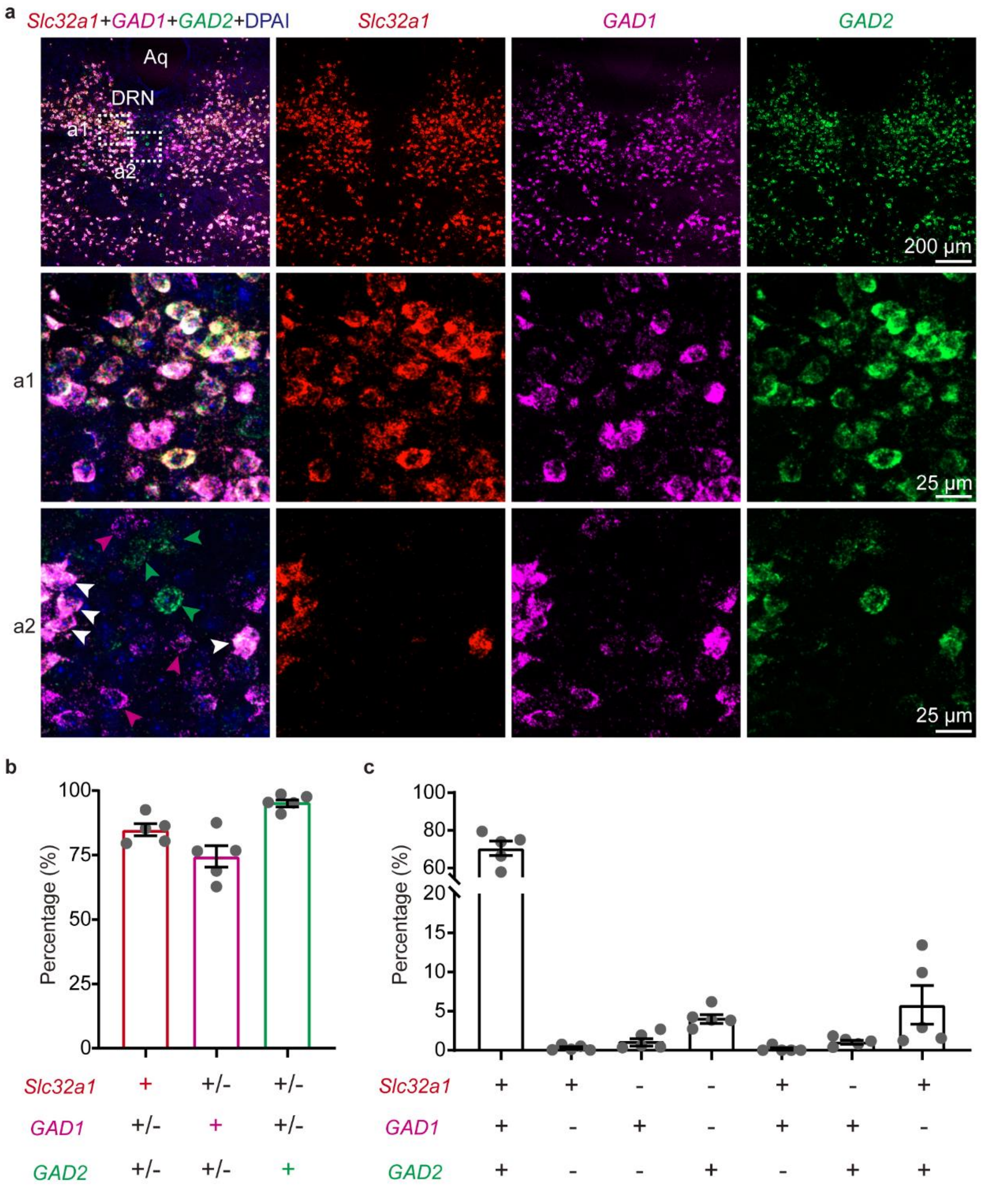

Supplementary Figure 15. The distribution of *Slc32a1*(*Vgat*), *GAD1*, and *GAD2* neurons in the DRN. a, Representative images showing the expression of *Slc32a1-, GAD1-*, and *GAD2*-positive neurons in the DRN. The zoomed area a1 shows that most DRN GABAergic neurons express all three markers, whereas the zoomed area a2 shows that DRN GABAergic neurons expressing one or two of GABAergic markers. The white arrowheads indicate *Slc32a1-, GAD1-*, and *GAD2*-triple-positive neurons. The purple arrowheads indicate *GAD1*-positive neurons. The greed arrowheads indicate *GAD2*-positive neurons. Aq, aqueduct. **b**, Percentage of *Slc32a1-, GAD1-*, and *GAD2*-positive neurons among all DRN GABAergic neurons. n=5 mice. **c**, Percentage of DRN GABAergic neurons expressing either *Slc32a1, GAD1*, or *GAD2.* n=5 mice. Data (**b**, **c**) are presented as mean ± SEM.

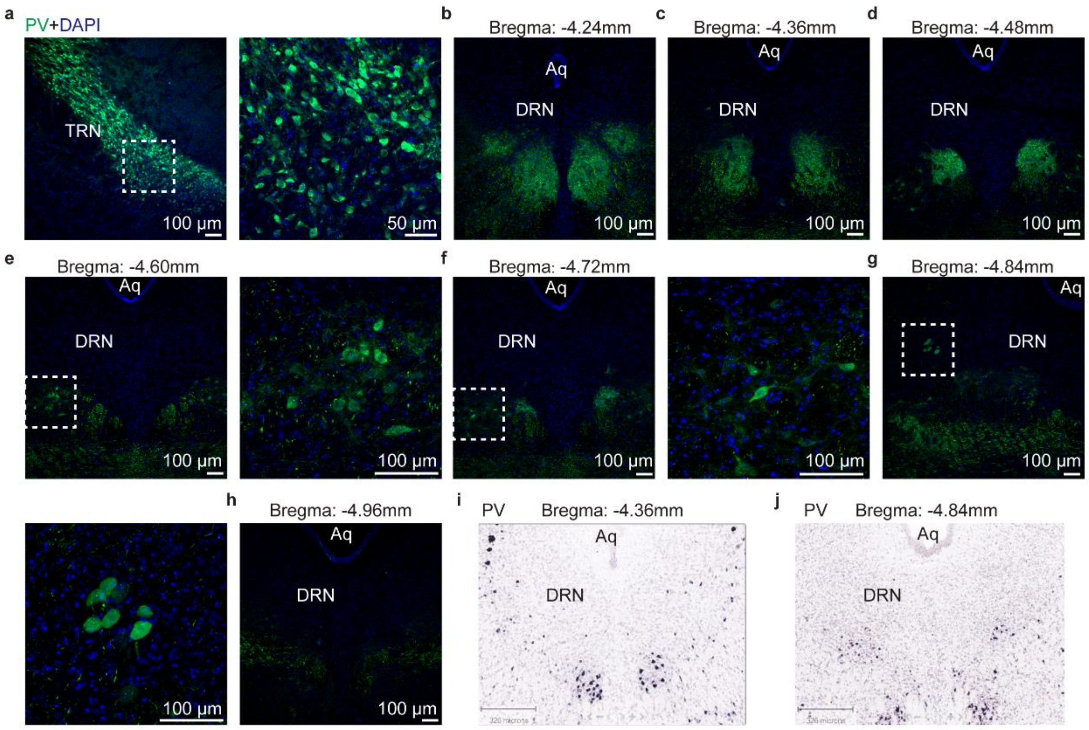

Supplementary Figure 16. The expression of parvalbumin (PV) neurons in the DRN and adjacent brain regions. a, Images showing the expression of PV-positive neurons in the thalamic reticular nucleus (TRN). Right image is an enlarged view of zoomed area in the left. **b** to **h**, PV-positive neurons in the DRN and adjacent brain regions. PV-positive neurons are rarely expressed in the DRN. Results were repeated in 3 mice. Aq, aqueduct. **i**, **j**, *In situ* hybridization with *PV* in the DRN from Allen Brain Atlas (*https://mouse.brain-map.org/experiment/show/868*).
