# Supplementary material for: A midbrain GABAergic circuit constrains wakefulness in a mouse model of stress
Source: Nat Commun. 2024 Mar 28;15:2722. doi: 10.1038/s41467-024-46707-9 (PMC10978901; doi:10.1038/s41467-024-46707-9)
Supplement: Supplementary file 3 — Description of Additional Supplementary Files [file 41467_2024_46707_MOESM3_ESM.pdf]

Description of Additional Supplementary Files

Supplementary Movie 1. Effects of optogenetic stimulation on wakefulness/sleep states of a DRN^{GAD2}-ChR2-mCherry mouse and a DRN^{GAD2}-mCherry mouse. Shown are EEG power spectrogram, EMG traces, color-coded hypnograms, and video recordings of a DRN^{GAD2}-ChR2-mCherry mouse and a DRN^{GAD2}-mCherry mouse. Laser stimulation induces a transition from wakefulness to NREM sleep in DRN^{GAD2}-ChR2-mCherry mouse, whereas has no obvious effects on behavioral states in DRN^{GAD2}-mCherry mouse. Laser stimulation period is indicated by blue stripe. The video is played at 4× the original speed. W, wake; NR, NREM.

Supplementary Movie 2. Effects of optogenetic inhibition of DRN^{GAD2} neurons on tail shock-induced increase of pupil size. Shown are pupil recordings and traces of pupil size from a DRN^{GAD2}-GtACR-GFP mouse. The vertical red dashed lines are the average of normalized pupil size from time -5s to 0s. Red arrows indicate the time points of tail shock (0.1 mA, 1s). Laser stimulation was simultaneously delivered with tail shock and lasted for 10s. $Pupil_{Norm.}$, normalized pupil size.
