# Supplementary material for: A midbrain GABAergic circuit constrains wakefulness in a mouse model of stress
Source: Nat Commun. 2024 Mar 28;15:2722. doi: 10.1038/s41467-024-46707-9 (PMC10978901; doi:10.1038/s41467-024-46707-9)
Supplement: Supplementary file 6 — Reporting Summary [file 41467_2024_46707_MOESM6_ESM.pdf]

nature portfolio

Corresponding author(s): Zhian Hu

Last updated by author(s): Jan 31, 2024

Reporting Summary

Nature Portfolio wishes to improve the reproducibility of the work that we publish. This form provides structure for consistency and transparency in reporting. For further information on Nature Portfolio policies, see our Editorial Policies and the Editorial Policy Checklist.

Statistics

For all statistical analyses, confirm that the following items are present in the figure legend, table legend, main text, or Methods section.

n/a	Confirmed	
☐	☒	The exact sample size (*n*) for each experimental group/condition, given as a discrete number and unit of measurement
☐	☒	A statement on whether measurements were taken from distinct samples or whether the same sample was measured repeatedly
☐	☒	The statistical test(s) used AND whether they are one- or two-sided<br>*Only common tests should be described solely by name; describe more complex techniques in the Methods section.*
☒	☐	A description of all covariates tested
☐	☒	A description of any assumptions or corrections, such as tests of normality and adjustment for multiple comparisons
☐	☒	A full description of the statistical parameters including central tendency (e.g. means) or other basic estimates (e.g. regression coefficient) AND variation (e.g. standard deviation) or associated estimates of uncertainty (e.g. confidence intervals)
☐	☒	For null hypothesis testing, the test statistic (e.g. *F*, *t*, *r*) with confidence intervals, effect sizes, degrees of freedom and *P* value noted<br>*Give P values as exact values whenever suitable.*
☒	☐	For Bayesian analysis, information on the choice of priors and Markov chain Monte Carlo settings
☒	☐	For hierarchical and complex designs, identification of the appropriate level for tests and full reporting of outcomes
☒	☐	Estimates of effect sizes (e.g. Cohen's *d*, Pearson's *r*), indicating how they were calculated

Our web collection on statistics for biologists contains articles on many of the points above.

Software and code

Policy information about availability of computer code

Data collection	1. EEG/EMG data were collected using Vital Recorder software (Kissei Comtec, Japan);<br>2. Fiber photometry and pupil recording data were collected using Fiber Photometry System (Inper Technology, China);<br>3. Patch-clamp recording data were collected using PatchMater (HEKA Elektronik);<br>4. Histological images were taken using LSM 800 confocal microscope (Carl Zeiss).
Data analysis	1. EEG/EMG data were analysis using SleepSign software (Kissei Comtec, Japan);<br>2. Fiber photometry data were analyzed using Inper Data Process (Inper Technology, China);<br>3. Pupil recording data were analyzed data using Bonsai (http://bonsai-rx.org/);<br>4. Patch-clamp recording data were analyzed using Pulse/Pulsefit v.8.74(HEKA Elektronik);<br>5. Histological results were analyzed using ZEN 2012 software or ImageJ software;<br>6. Statistical analyses were performed using SigmaPlot 14.0. GraphPad Prism 8.0, and Matlab 2021a;<br>7. Code for locomotor activity analysis in open filed test are available on GitHub (https://github.com/shuanchengren/open-filed-analysis).

For manuscripts utilizing custom algorithms or software that are central to the research but not yet described in published literature, software must be made available to editors and reviewers. We strongly encourage code deposition in a community repository (e.g. GitHub). See the Nature Portfolio guidelines for submitting code & software for further information.

Data

Policy information about availability of data

All manuscripts must include a data availability statement. This statement should provide the following information, where applicable:

- Accession codes, unique identifiers, or web links for publicly available datasets
- A description of any restrictions on data availability
- For clinical datasets or third party data, please ensure that the statement adheres to our policy

The complete raw data from EEG/EMG recording, fiber photometry recording, behavioral experiments, and histological experiments are huge and presented in diverse nature and formats. These data are available upon request from the corresponding author. No datasets that require mandatory deposition into a public database were generated in the current study. Source data underlying Figs. 1-7 and Supplementary Figures are available as a Source Data file and shared in https://doi.org/10.6084/m9.figshare.24779127. Source data are provided with this paper.

Research involving human participants, their data, or biological material

Policy information about studies with human participants or human data. See also policy information about sex, gender (identity/presentation), and sexual orientation and race, ethnicity and racism.

Reporting on sex and gender	N/A
Reporting on race, ethnicity, or other socially relevant groupings	N/A
Population characteristics	N/A
Recruitment	N/A
Ethics oversight	N/A

Note that full information on the approval of the study protocol must also be provided in the manuscript.

Field-specific reporting

Please select the one below that is the best fit for your research. If you are not sure, read the appropriate sections before making your selection.

☒ Life sciences ☐ Behavioural & social sciences ☐ Ecological, evolutionary & environmental sciences

For a reference copy of the document with all sections, see nature.com/documents/nr-reporting-summary-flat.pdf

Life sciences study design

All studies must disclose on these points even when the disclosure is negative.

Sample size	Sample sizes were chosen based upon previous publications using optogenetic and chemogenetic tools for the study of the sleep–wake circuitry (A. Eban-Rothschild et al., Nat. Neurosci., 2017; J. R. Cho et al., Neuron,2017).
Data exclusions	If post experiment histology show that the viral expression or fiber location is outside the targeted brain area, data from that mouse were excluded.
Replication	All EEG/EMG recording, fiber photometry recording, electrophysiological recording and behavioral experiments were independently performed over at least 5 replicates. Histological experiments were performed over at least 3 replicates. The study effects were all successfully replicated in the experiments.
Randomization	All samples were randomly allocated into each group.
Blinding	Investigators were blinded to the group allocation during data acquisition and data analysis.

Reporting for specific materials, systems and methods

We require information from authors about some types of materials, experimental systems and methods used in many studies. Here, indicate whether each material, system or method listed is relevant to your study. If you are not sure if a list item applies to your research, read the appropriate section before selecting a response.

Materials & experimental systems

n/a	Involved in the study
☐	☒ Antibodies
☒	☐ Eukaryotic cell lines
☒	☐ Palaeontology and archaeology
☐	☒ Animals and other organisms
☒	☐ Clinical data
☒	☐ Dual use research of concern
☒	☐ Plants

Methods

n/a	Involved in the study
☒	☐ ChIP-seq
☒	☐ Flow cytometry
☒	☐ MRI-based neuroimaging

Antibodies

Antibodies used	Primary antibodies: rabbit polyclonal anti-TH (1:1000, Abcam, ab112), Rabbit monoclonal anti-TPH2 (clone,EPR19191)(1:500, Abcam ab184505); rabbit monoclonal anti-parvalbumin (1:500, Abcam, Ab181086)<br>Secondary antibodies: donkey anti-rabbit Alexa Fluor 488 (1:500; A21207; Invitrogen)
Validation	Rabbit polyclonal anti-TH (Abcam, ab112): https://www.abcam.cn/products/primary-antibodies/tyrosine-hydroxylase-antibody-neuronal-marker-ab112.html<br>Rabbit monoclonal anti-TPH2 (clone,EPR19191)(Abcam ab184505): https://www.abcam.cn/products/primary-antibodies/tph2-antibody-epr19191-ab184505.html<br>Rabbit monoclonal anti-parvalbumin (Abcam, Ab181086):https://www.abcam.cn/products/primary-antibodies/parvalbumin-antibody-epr13091-ab181086.html

Animals and other research organisms

Policy information about studies involving animals; ARRIVE guidelines recommended for reporting animal research, and Sex and Gender in Research

Laboratory animals	In all experiments, adult (8-12 weeks, 22-26g) male or female GAD2-IRES-Cre mice (The Jackson Laboratory, stock number: 010802), vGlut2-IRES-Cre mice (The Jackson Laboratory, stock number: 028863), and WT C57BL/6 mice were used. Mice were housed at controlled environmental temperature (22±1°C), humidity (~50%), and 12h light/12h dark cycle (light on between 6 am (ZT0) and 6 pm). Mice were group (2-5)-housed with ad libitum access to food and water.
Wild animals	No wild animals were used in this study.
Reporting on sex	Male or female mice were used in this study.
Field-collected samples	No field collected samples were used in this study.
Ethics oversight	All experimental protocols are in accordance with Army Medical University Guide for the Care and Use of Laboratory Animals.

Note that full information on the approval of the study protocol must also be provided in the manuscript.

Plants

Seed stocks	No plants were used in this study.
Novel plant genotypes	No plants were used in this study.
Authentication	No plants were used in this study.
